# Novel strategy for disease risk prediction incorporating predicted gene expression and DNA methylation data: a multi‐phased study of prostate cancer

**DOI:** 10.1002/cac2.12205

**Published:** 2021-09-14

**Authors:** Chong Wu, Jingjing Zhu, Austin King, Xiaoran Tong, Qing Lu, Jong Y. Park, Liang Wang, Guimin Gao, Hong‐Wen Deng, Yaohua Yang, Karen E. Knudsen, Timothy R. Rebbeck, Jirong Long, Wei Zheng, Wei Pan, David V. Conti, Christopher A Haiman, Lang Wu

**Affiliations:** ^1^ Department of Statistics Florida State University Tallahassee FL 32304 USA; ^2^ Cancer Epidemiology Division, Population Sciences in the Pacific Program, University of Hawaii Cancer Center University of Hawaii at Manoa Honolulu HI 96813 USA; ^3^ Department of Epidemiology and Biostatistics Michigan State University East Lansing MI 48824 USA; ^4^ Department of Biostatistics University of Florida Gainesville FL 32603 USA; ^5^ Department of Cancer Epidemiology H. Lee Moffitt Cancer Center and Research Institute Tampa FL 33612 USA; ^6^ Department of Tumor Biology H. Lee Moffitt Cancer Center and Research Institute Tampa FL 33612 USA; ^7^ Department of Public Health Sciences University of Chicago Chicago IL 60637 USA; ^8^ Center of Bioinformatics and Genomics, Department of Global Biostatistics and Data Science Tulane University New Orleans LA 70112 USA; ^9^ Division of Epidemiology, Department of Medicine, Vanderbilt Epidemiology Center, Vanderbilt‐Ingram Cancer Center Vanderbilt University Medical Center Nashville TN 37203 USA; ^10^ Department of Cancer Biology, Sidney Kimmel Cancer Center Thomas Jefferson University Philadelphia PA 19107 USA; ^11^ Department of Medical Oncology Dana‐Farber Cancer Institute Boston MA 02215 USA; ^12^ Department of Epidemiology Harvard TH Chan School of Public Health Boston MA 02115 USA; ^13^ Division of Biostatistics University of Minnesota Minneapolis MN 55455 USA; ^14^ Department of Preventive Medicine Keck School of Medicine University of Southern California/Norris Comprehensive Cancer Center Los Angeles CA 90033 USA

**Keywords:** risk prediction, polygenic risk scores, predicted gene expression, predicted DNA methylation, integrative models, prostate cancer

## Abstract

**Background:**

DNA methylation and gene expression are known to play important roles in the etiology of human diseases such as prostate cancer (PCa). However, it has not yet been possible to incorporate information of DNA methylation and gene expression into polygenic risk scores (PRSs). Here, we aimed to develop and validate an improved PRS for PCa risk by incorporating genetically predicted gene expression and DNA methylation, and other genomic information using an integrative method.

**Methods:**

Using data from the PRACTICAL consortium, we derived multiple sets of genetic scores, including those based on available single‐nucleotide polymorphisms through widely used methods of pruning and thresholding, LDpred, LDpred‐funt, AnnoPred, and EBPRS, as well as PRS constructed using the genetically predicted gene expression and DNA methylation through a revised pruning and thresholding strategy. In the tuning step, using the UK Biobank data (1458 prevalent cases and 1467 controls), we selected PRSs with the best performance. Using an independent set of data from the UK Biobank, we developed an integrative PRS combining information from individual scores. Furthermore, in the testing step, we tested the performance of the integrative PRS in another independent set of UK Biobank data of incident cases and controls.

**Results:**

Our constructed PRS had improved performance (C statistics: 76.1%) over PRSs constructed by individual benchmark methods (from 69.6% to 74.7%). Furthermore, our new PRS had much higher risk assessment power than family history. The overall net reclassification improvement was 69.0% by adding PRS to the baseline model compared with 12.5% by adding family history.

**Conclusions:**

We developed and validated a new PRS which may improve the utility in predicting the risk of developing PCa. Our innovative method can also be applied to other human diseases to improve risk prediction across multiple outcomes.

## Graphical abstract

An integrative score incorporating genetically predicted gene expression and DNA methylation and other genomic and non‐genomic information advanced our understanding of using genomic information to stratify subjects for prostate cancer.

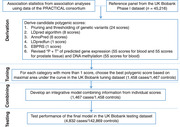

## 1 BACKGROUND

Prostate cancer (PCa) is the most commonly diagnosed type of cancer and the second leading cause of cancer deaths for men in the United States [1]. Due to the huge public health burden caused by this disease, stratifying men based on their risk of PCa is critical to improving screening strategies. The etiology of PCa is poorly understood, with only a few established risk factors identified such as age, race/ethnicity, and family history [2]. Epidemiological studies have suggested that genetic factors play an essential role in the etiology of PCa [3]. Thus, comprehensive information derived from genetic factors may contribute to PCa risk stratification.

Over the last decade, genome‐wide association studies (GWAS) have identified 269 PCa susceptibility variants [4, 5]. As an approach to identify men of high risk from the general population, previous studies have attempted to construct polygenic risk scores (PRS) by leveraging known PCa risk single‐nucleotide polymorphisms (SNPs), which showed promising performance [4, 5]. However, existing PRSs remain unsatisfactory for providing a comprehensive assessment of the potential of genomic information in predicting PCa risk for the following reasons. First, GWAS‐identified susceptibility variants only explain less than one‐half of the familial PCa risk [4, 5]. Second, previous studies did not apply comprehensive and state‐of‐the‐art statistical methods in developing disease prediction models [4, 5]. Instead, these studies have largely relied only on relatively intuitive statistical methods (e.g., using GWAS‐identified risk variants to develop PRS) that may not fully capture the predictive value of genomic information. Third, to date, most of the existing studies have largely lacked testing via independent cohorts. Ideally, an independent dataset is crucial for assessing the performance of PRSs in an unbiased way.

It is expected that more informative PRSs incorporating genetic factors beyond GWAS‐identified risk variants would improve the performance of a risk prediction model. Studies suggest that methods such as pruning and thresholding (P + T) [6] and the LDpred algorithm [7] could facilitate the development of comprehensive PRSs for common diseases. Using these methods, PRSs are constructed by summing the cumulative effect of many (or even all) genetic variants and could identify a substantially larger fraction of the population than found by rare monogenic mutations at comparable disease risk [8]. However, a more recent work testing performance in incident disease cases reported that the addition of a PRS developed using such methods could only modestly improve the predictive accuracy in diseases such as coronary artery disease, thus, limiting their clinical utility [9]. It remains largely unknown whether these observations are indicative of the limited clinical potential of PRS, or whether the current observations are due to the fact that available modern PRS methods such as P + T and LDpred may still be suboptimal to capture the full potential of genetic factors. For example, it remains largely unknown whether the information of DNA methylation and gene expression, which are known to be directly involved in the etiology of many diseases including PCa, could improve PRS performance that are not captured by existing methods. Recently, we conducted novel transcriptome‐wide association studies [10] and methylome‐wide association studies [11], and identified multiple genes and DNA methylation biomarker candidates with genetically predicted levels to be associated with PCa risk. Such strategies discovered novel PCa‐associated genes and methylation biomarkers, many of which are distant from GWAS‐identified risk variants. Importantly, we identified that a proportion of such significant associations tended to be independent of GWAS‐identified risk variants [10, 11]. These novel and independent signals indicate that imputed gene expression and/or methylation may provide additional and independent information beyond genetic variants for disease prediction. Based on the theory developed in causal inference [12], using genetically imputed gene expression and methylation can overcome unmeasured confounding factors (such as environmental factors) and yield more accurate estimations of the effects for each gene and CpG site, potentially providing more robust and generalizable PRS. Thus, we hypothesized that incorporating information of genetically imputed gene expression and DNA methylation may significantly improve predictive performance. Further, we hypothesized that an integrative PRS combining information from multiple different PRS methods can further improve disease prediction performance.

In the current study, we incorporated genetically imputed gene expression and DNA methylation information using a newly revised P + T method and combined information from this new method and several existing methods to build a comprehensive PCa risk prediction model. We developed new prediction models for PCa risk using the PRACTICAL/ELLIPSE consortia GWAS dataset [4], a very large genetic dataset for PCa risk. We further used independent datasets from the UK Biobank for model tuning and testing.

## 2 MATERIALS AND METHODS

### 2.1 Genetic prediction models for gene expression of normal prostate and whole blood tissues

The detailed information for normal prostate tissue and blood gene expression prediction models were as previously described [13]. In brief, a joint‐tissue imputation approach that borrows information across different tissues and leverages shared genetic regulatory effects was used to develop these tissue‐specific prediction models. These prediction models were developed by leveraging The Genotype‐Tissue Expression v8 data and downloaded from Zenodo (https://doi.org/10.5281/zenodo.3842289).

### 2.2 Whole‐blood DNA methylation genetic prediction models

Details for the whole‐blood DNA methylation prediction models were as previously described [14]. Briefly, the genetic and blood DNA methylation data from the BIOS Consortium (containing 4,008 samples; http://wiki.bbmri.nl/wiki/BIOS_start‐/) were used for the model building. For each CpG site with a significant methylation quantitative trait locus, a lasso was fit using glmnet with in‐cis SNPs closer than 250 kb as candidate predictors to predict DNA methylation levels.

### 2.3 Associations of genetically predicted gene expression and DNA methylation levels with PCa risk

We evaluated associations of genetically predicted gene expression and DNA methylation levels with PCa risk by using the S‐PrediXcan method [15]. We leveraged summary statistics data for GWAS of 79,194 PCa cases and 61,112 controls in the consortia PRACTICAL, CRUK, CAPS, BPC3, and PEGASUS [4]. The PCa risk GWAS summary data were also used for risk prediction model derivation. Briefly, these included data from several GWAS: UK stage 1 (1,854 cases/1,894 controls) and UK stage 2 (3,650 cases/3,940 controls), CaPS 1 (474 cases/482 controls) and CaPS 2 (1,458 cases/512 controls), BPC3 (2,068 cases/2,993 controls), NCI PEGASUS (4,600 cases/2,941 controls), iCOGS (20,219 cases/20,440 controls), and OncoArray (46,939 cases and 27,910 controls). The genotype data were imputed using the June 2014 release of the 1000 Genomes Project data as a reference. Logistic regression summary statistics were then meta‐analyzed using an inverse variance fixed‐effect approach.

The *Z* score for the associations between predicted gene expression/DNA methylation levels and PCa risk was estimated based on the formula

$$Z_{m}\approx \;\sum_{s\in \mathrm{Mode}l_{m}}w_{sm}\frac{{\hat{\sigma }}_{s}}{{\hat{\sigma }}_{m}}\;\frac{{\hat{\beta }}_{s}}{\mathrm{se}({\hat{\beta }}_{s})}.$$

Here $w_{sm}$ represents the weight of the SNP s on the expression/DNA methylation levels of the gene/CpG m. ${\hat{\beta }}_{s}$ and $\mathrm{se}({\hat{\beta }}_{s})$ refer to the GWAS‐estimated effect size and standard error of the SNP s on PCa risk, respectively. ${\hat{\sigma }}_{s}$ and$\;{\hat{\sigma }}_{m}$ are the estimated variances of the SNP s and the predicted expression/methylation level at the gene/CpG m, respectively.

### 2.4 UK Biobank data

UK Biobank study is a large prospective cohort study that covers a wide range of complex diseases and enrolls individuals aged 40‐69 years across the United Kingdom, starting in 2006. Details of the UK Biobank study are as previously described [16]. This study was approved by the UK Biobank for using its data. Approval from UK Biobank's research ethics committee grants that approved researchers do not need separate ethics approval. PCa cases in the UK Biobank were selected by combining hospital episode statistics (HES) data and self‐reported data. Specifically, cases were defined as hospital admission (data fields 41202, 41203, 41270, and 41271), type of cancer (data fields 40006 and 40013), cause of death (data fields 40001 and 40002) due to ICD‐9 185.9 or ICD‐10 C61, or cancer code, self‐reported (data field 20001). Non‐PCa individuals were defined as the control population. To reduce biological misclassification, we excluded individuals from the controls if they have secondary malignant neoplasm (ICD‐9 198.8 or ICD‐10 C79.8) defined in hospital admission, type of cancer, or cause of death data or if they have PCa‐related procedures (operation code 1208 or OPCS‐4 M708, M718, Y123, Y132, Y53, and Z422), leaving 2,925 males in the tuning dataset, 2,925 males in the combining dataset, and 147,701 males in the testing dataset. For the age of event, we used the smaller value of self‐reported age and calculated age based on the earliest hospital record for the event. Prevalent case was defined as the case with the age of event (i.e., having PCa) preceding the age at recruitment. Follow‐up time for each participant was calculated as the number of years from assessment date until either event of interest (having PCa), or competing event (other causes of death), or censorship date (January 1, 2019).

We applied standard quality‐control procedures to the genotype data in the UK Biobank. Briefly, we downloaded Version 3 of the Imputed Genotypes data from the UK Biobank and restricted our analyses to autosomal genetic variants. We kept variants with minor allele frequency (MAF) > 1%, imputation information score > 0.3, genotype missing rate < 10%, and Hardy‐Weinberg equilibrium (HWE) *P* > 10^–10^. Genetic variants with ambiguous strands (A/T or C/G) were removed. We followed the guideline from the UK Biobank [17] and extracted the maximum unrelated White British individuals, which were defined as individuals with no relative 3^rd^ degree or closer. We further excluded outliers due to heterozygosity or genotype missing rates (>2% missing rate) and individuals with discordance for the reported versus genotypic inferred sex or withdrawal of informed consent. We focused on independent White British individuals to avoid potential relatedness between the validation and testing datasets [18]. Population stratification was controlled via adjusting for the genetic principal components provided by the UK Biobank [17].

### 2.5 PCa risk prediction model building, validating, and testing

Of note, the validation, combining, and testing datasets can be viewed as approximately independent datasets as we only used the unrelated White British individuals.

Multiple sets of genetic scores were derived for each individual, including 1) existing PRSs based on all available SNPs, constructed by widely used methods P + T (PRSice‐2) [6], LDpred [7], LDpred‐funt [19], AnnoPred [20], and EBPRS [21], 2) Expression‐PRSs constructed using the genetically predicted gene expression levels in prostate tissues and blood, and 3) Methylation‐PRSs constructed using genetically predicted DNA methylation levels in the blood. For the existing PRSs using SNPs, the P + T strategy involves LD pruning and subsequently aggregating SNPs that exceed a specified significance level in the GWAS, while LDpred infers the expected regression coefficient of SNP by considering LD among SNPs. LDpred‐funt, AnnoPred, and EBPRS incorporate different types of functional information when constructing the PRSs. We followed a standard pipeline for constructing a set of candidate PRSs using the P + T (by PRSice‐2) and LDpred strategies [8] and used default settings recommended in LDpred‐funt, AnnoPred, and EBPRS. Briefly, the PRSs based on P + T were created over a range of *P*‐value threshold ($1.0,\;0.5,\;0.05,\;5\times 10^{-4},\;5\times 10^{-6},\;\mathrm{and}\;5\times 10^{-8})$ and *r* ^2^ (0.2, 0.4, 0.6, and 0.8); the PRSs based on LDpred were created over a range of ρ values (the fraction of causal variants; 1.0, 0.3, 0.1, 0.03, 0.01, 0.003, and 0.001); the LDpred‐funt was based on the hapmap3 subset; AnnoPred was based on the three different tiers of functional information (tier0, tier1, tier3; tier2 was not used due to convergence problems) and two types of priors (h2, pT); EBPRS only provided one PRS, with no need for tuning. For genetic scores of predicted gene expression or DNA methylation levels, we used the formula $wGRS=\sum_{j=1}^{p}\beta_{j}SNP_{j}$, with $\beta_{j}$ representing the regression coefficient of the *j*th SNP for the corresponding gene expression level/DNA methylation level. Specifically, we proposed a revised P + T strategy in which we calculated several scores by clumping correlated predicted DNA methylation/gene expression levels and aggregating the effects that exceed a specified significance level. In other words, we replaced SNPs with predicted gene expression/methylation levels in the revised P + T strategy. In brief, predicted DNA methylation/gene expression levels were first clumped (P step) so that only predicted DNA methylation/gene expression levels that are weakly or not correlated with each other were retained. Clumping was iteratively cycled through all available genes/CpG sites, starting with those with the smallest *P* value. Each clump contained all genes/CpG sites in the same chromosome of the index gene/CpG site that were correlated with the index gene/CpG site beyond a particular correlation threshold (*r* ^2^). Only the most significant gene/CpG site in each linkage disequilibrium‐based clump across the genome were kept. Next, we removed genes/CpG sites with a *P* value higher than a particular threshold (T step). In the end, we built a PRS as the weighted summation of the selected predicted gene expression/DNA methylation levels with association estimate betas as weights. PRSs were created over a range of *P* values ($5\times 10^{-8},\;5\times 10^{-6},\;5\times 10^{-4},\;0.05,\;0.5,$ and 1) and *r* ^2^ thresholds (0.2, 0.4, 0.6, and 0.8). We used the same tuning parameters used in other studies [8, 9] for the standard P + T method.

In the discovery step, we used the PCa GWAS summary statistics to calculate PRSs by the methods described above. We divided the case‐control sample of 2,925 prevalent PCa cases and 2,925 randomly selected controls in the UK Biobank into two almost equal‐sized subsets for tuning scores and combining scores. In the tuning step, we used a case‐control sample of 1,458 prevalent PCa cases and 1,467 controls as tuning data to select the PRSs with the best performance, defined as the maximal area under the receiver operator characteristic curve (AUC). Specifically, for each of the tested methods (when there were more than one candidate score), we selected the best score with the maximal AUC in a logistic regression model, in which PCa status was the outcome and baseline variables [including age, first ten genetic principal components (PCs), and genotype array] and constructed PRS were covariates. Considering that different methods may capture different types of information, to fully capture the potential of the PRS, we used another independent case‐control sample of 1,467 prevalent PCa cases and 1,458 controls to develop an integrative PRS combining information from individual scores using logistic regression. Briefly, we regressed the PCa status on the 8 PRSs constructed by different methods and baseline covariates. We then constructed the combined PRS by $GRS=\sum_{j=1}^{q}{\hat{\beta }}_{j}PRS_{j}$, where ${\hat{\beta }}_{j}$ is the estimated coefficient of $PRS_{j}$ in the logistic regression.

In the testing step, we tested the performance of the integrative PRS in an independent UK Biobank data of 4,832 incident cases and 142,869 controls. Of note, we used this design to maximize available incident cases in the testing step. We also assessed the performance of individual PRSs in this testing dataset for comparison with our integrative PRS. Primarily, the discrimination of each model was assessed using Harrell's C statistic. The C statistic is a rank‐order statistic for predictions against true outcomes, with values ranging from 0.5 (no discrimination) to 1.0 (perfect discrimination). To achieve unbiased evaluations, several additional criteria including AUC, Nagelkerke's pseudo‐R^2^ metric, and area under the precision‐recall curve (AUPRC) were used.

Cox proportional hazards model was used to estimate hazard ratios (HRs) and to calculate 10‐year or 5‐year PCa event probabilities. The proportionality assumption was graphically inspected by scaled Schoenfeld residuals. We graphically assessed the calibration of the original models and their subsequent recalibration by plotting the mean predicted cumulative incidence against the observed cumulative incidence within tenths of the predicted cumulative incidence. The calibration slope and the Greenwood‐Nam‐D'Agostino test [22] were used to quantitatively assess the calibration of models. For recalibration, we estimated the baseline survival function in the cohort (through the basehaz function in R) and combined this with the predicted HRs from the validation dataset as a covariate in a Cox model to obtain recalibrated predicted probabilities. For discrimination, we examined the difference in C statistics when the PRS was added to the baseline model and evaluated the significance of the difference of C statistic by a one‐shot nonparametric approach [23]. The net reclassification improvement (NRI) was applied to assess the correct reassignment among risk categories [24]. Following previous works [8, 25], individuals were then binned into 100 groupings based on the percentile of PRS, and the absolute risk of PCa within each bin was determined. All statistical analyses were conducted using the R software (The Comprehensive R Archive Network, version 3.6.0, https://cran.r‐project.org).

## 3 RESULTS

### 3.1 PRS derivation, tuning, and combining

**FIGURE 1:**
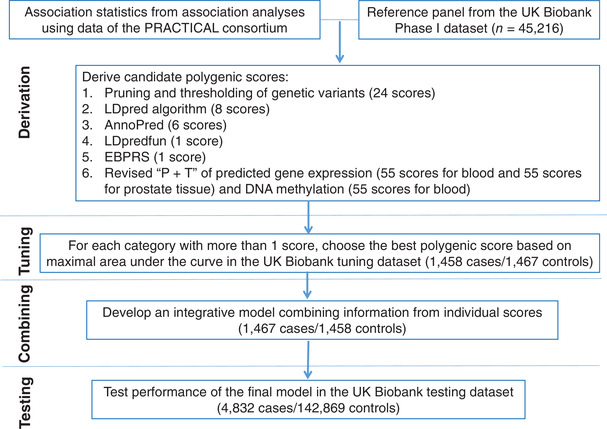
Study design and workflow. Multiple sets of genome‐wide polygenic risk scores (PRSs) were derived by combining summary association statistics from association studies using data of the PRACTICAL consortium and a reference panel of 45,216 males in the UK Biobank Phase I dataset. Candidate PRSs were derived using six strategies: 1) pruning and thresholding (P + T)– aggregation of independent polymorphisms that exceed a specified level of significance in the discovery genome‐wide association study (GWAS) (24 candidates); 2) LDpred computational algorithm, a Bayesian approach to calculate a posterior mean effect for all variants based on a prior (effect size in the prior GWAS) and subsequent shrinkage based on linkage disequilibrium (8 candidates); 3) AnnoPred (6 candidates); 4) LDpredfun (1 score); 5) EBPRS (1 score); and 6) revised P + T approach incorporating predicted gene expression (55 candidates for blood and 55 scores for prostate tissue) and DNA methylation (55 candidates for blood). For each of the above categories, the optimal PRS was chosen based on the area under the receiver‐operator curve (AUC) in the UK Biobank tuning dataset (1,458 prevalent cases and 1,467 controls). We then derived the integrative model combining information from constructed scores (1,467 prevalent cases and 1,458 controls). We subsequently tested the model performance in an independent UK Biobank testing dataset (4,832 incident cases and 142,869 controls)

The overall study design is shown in Figure 1. In the tuning step, using the UK Biobank tuning dataset of 1,458 prevalent cases and 1,467 controls, the AUC of each of the scores were estimated (Supplementary Table S1). For each category containing more than one candidate score, we chose the best score showing the highest AUC. In another UK Biobank dataset of 1,467 prevalent cases and 1,458 controls, we developed an integrative PRS combining information from each score derived from the six tested methods. Based on checking potential collinearity issues in this step, the PRS constructed by different tested methods tended not to be highly correlated (Supplementary Figure S1).

### 3.2 Testing of the integrative PRS

To achieve unbiased and robust results, we used an independent testing dataset (*n* = 147,701) for the downstream analyses. This dataset comprises 4,832 incident PCa cases and 142,869 non‐PCa individuals. The mean age was 57.0 years, and the median follow‐up time for PCa cases was 4.7 years (interquartile range, 3.9 years). We examined the association of PRSs with incident PCa cases.

**FIGURE 2:**
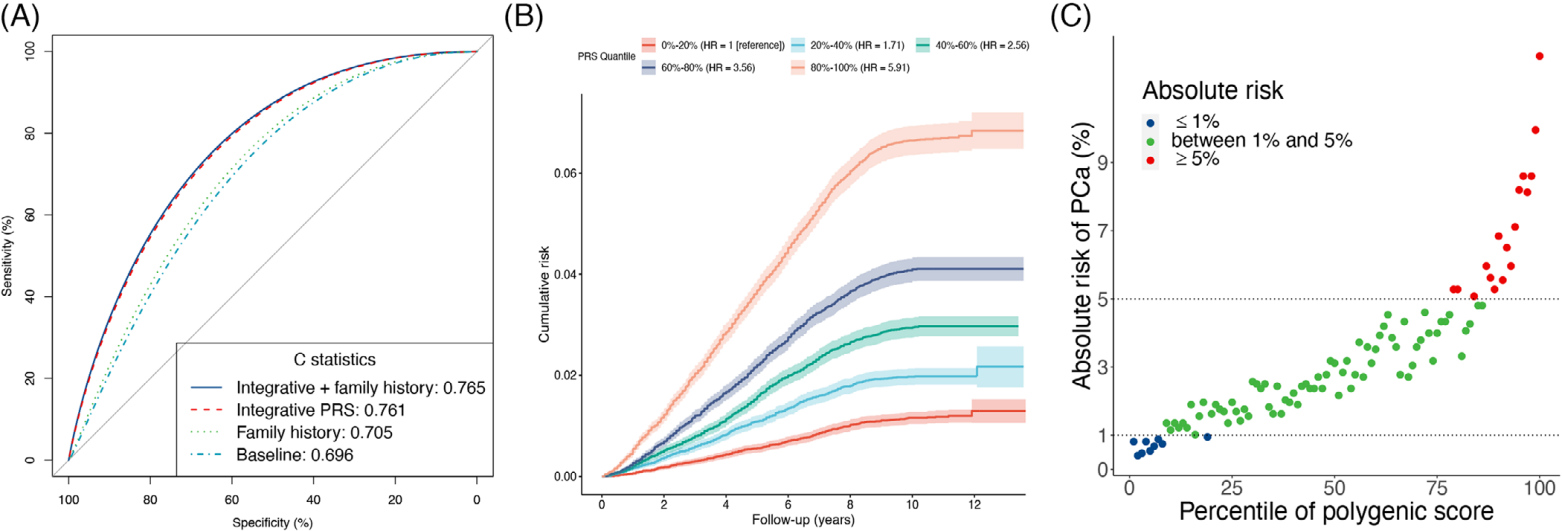
Polygenic risk score assessment with incident cases. (A) Receiver operator characteristic curves and C statistics for different models in the independent testing dataset of 147,701 participants with 4,832 incident prostate cancer events. (B) The cumulative absolute risk of developing prostate cancer by quantiles of the overall polygenic score. The absolute risk was calculated based on UK incidence and mortality data and using the PRS relative risks estimated as described in the Material and Methods. The shaded part is 95% confidence interval. (C) The absolute risk of prostate cancer according to 100 groups of the testing cohort binned according to the percentile of the integrative polygenic risk score

Our novel integrative strategy achieved an unparalleled C statistic of 0.761 [95% confidence interval (CI), 0.755‐0.767], significantly higher than that of the baseline model which included age, genotype array, and population stratification (C statistic, 0.696; 95% CI, 0.690‐0.702) (Figure 2A and Supplementary Table S2). In other words, the addition of our integrative PRS to the baseline model showed a significant improvement in discrimination, with an associated change of 0.065 for C statistic (*P* < 0.001). Furthermore, by integrating information from different PRS methods, our final synthesized PRS performed much better than the scores derived using each individual benchmark method (P + T [26], LDpred [7], LDpred‐funt [19], AnnoPred [20], and EBPRS [21]). This was also true for several other widely used criteria for evaluating model performance, including AUC, pseudo‐R2 metric, and AUPRC (Supplementary Table S2). Our novel strategy demonstrated superior performance than PRSs derived using benchmark methods. As a result, for the downstream analyses, we focused on the PRS constructed by our novel integrative strategy.

### 3.3 PRS recalibration

When the observed and predicted cumulative incidences of PCa events were compared across each tenth of predicted risk, PRS plus baseline model overestimated risk across the ranges of predicted probabilities. To remedy this, following others’ works [9, 27], we recalibrated the model by fitting the predicted log‐HRs as covariates in the model, which substantially improved the model calibration (Supplementary Figure S2).

### 3.4 Evaluation of the integrative PRS for risk assessment of PCa

Next, we investigated the potential role of our developed PRS in the risk assessment of PCa. We first evaluated the HR in a Cox regression. The PRS had an adjusted HR of 2.01 (95% CI: 1.95‐2.07) per standard deviation increase of PRS (*P* < 0.001). Individuals in different quintiles of the PRS had significantly different risks (Figure 2B). For example, individuals in the top quintile of the PRS were at 5.91‐fold (95% CI: 5.27‐ to 6.62‐fold) risk of PCa than those in the bottom quintile (Figure 2B). Furthermore, the median percentile of the PRS for PCa cases and non‐PCa controls were 73 and 50, respectively. The absolute risk of PCa increased sharply in the right tail of polygenic score distribution, from 0.8% in the lowest percentile to 12.1% in the highest percentile (Figure 2C).

### 3.5 Comparison of the PRS and family history in risk assessment of PCa

In the end, we compared the risk assessment power between our developed PRS and family history. We first divided the participants in the test cohort into tertiles based on the predicted 10‐year risk from the baseline model: 7.9% of participants were reclassified to a higher risk group, and 25.5% of participants were reclassified to a lower risk group after adding PRS to the baseline model; in contrast, only 2.7% of participants were reclassified to a higher risk group, and 5.1% of participants were reclassified to a lower risk group after adding family history to the baseline model. Further, defining NRI in the continuous case, when the PRS for PCa was added to the baseline model, the predicted 10‐year risk changed by more than 1% for 44.5% of participants and changed by 5% or more for 6.4% of participants (Supplementary Figure S3). The overall NRI was 69.0% (95% CI, 64.9%–70.5%) (Table 1). In comparison, when family history was added to the baseline model, the predicted 10‐year risk changed by more than 1% for 5.3% of participants and changed by 5% or more of 0.15% participants. The increase in risk difference between cases and non‐cases (overall NRI) was 12.5% (95% CI, 11.3%–16.5%) (Table 1). When defining NRI in the categorical case, similar finding was observed for 5‐year risk prediction (Supplementary Table S3). These suggest that the PRS developed using our novel integrative strategy had much higher risk assessment power than family history.

**TABLE 1:** Net reclassification improvement (NRI) of the developed polygenic risk score and family history in predicting the risk of PCa

Population	No. of subjects	Five‐year risk		Ten‐year risk	
		NRI for PRS (95% CI)	NRI for family history (95% CI)	NRI for PRS (95% CI)	NRI for family history (95% CI)
PCa cases	4,832	0.294 (0.283 to 0.324)	−0.711 (−0.731 to −0.686)	0.266 (0.234 to 0.277)	−0.717 (−0.731 to −0.659)
Non‐cases	142,869	0.419 (0.411 to 0.435)	0.84 (0.837 to 0.841)	0.423 (0.400 to 0.434)	0.842 (0.824 to 0.844)
Full population	147,701	0.713 (0.697 to 0.756)	0.129 (0.109 to 0.151)	0.690 (0.649 to 0.705)	0.125 (0.113 to 0.165)

The NRI (in continuous case) of 5‐ and 10‐year risk was calculated by adding the PRS or family history to the baseline model.

PCa, prostate cancer; CI, confidence interval.

## 4 DISCUSSION

We developed a revised P + T method to incorporate genetically imputed gene expression and DNA methylation into PRS. Our integrative PRS achieved an unparalleled C statistic of 0.761 in an independent testing dataset of PCa cases. The PRS developed using our novel integrative strategy showed much higher risk assessment power than family history.

In recent years, there have been increasing interests in applying PRS for risk prediction of multiple human diseases [8, 28]. Despite the successes of earlier studies evaluating PRS performance in datasets including both prevalent and incident disease cases, some subsequent studies raised concerns for the clinical utility of such PRSs [9, 27, 29]. Indeed, when evaluating the performance of such PRSs focusing on incident cases only, there was limited evidence supporting the potential clinical utility of such newly developed PRS, such as in coronary artery disease [9, 27, 29]. It remains unknown whether the current observations of the less encouraging utility of PRS in disease risk prediction are due to the disadvantages of currently available PRS methods which largely miss potentially additional functional information, such as genetically predicted intermediate molecular levels. In this current work, we developed a new method to capture such information of genetically predicted gene expression and DNA methylation levels. The current work suggests that the integrative PRS incorporating such information and information captured from other PRS methods could substantially improve prediction accuracy to a greater extent than PRSs developed using individual existing methods and that with family history information. The newly proposed method is computationally efficient. For one chromosome and a tuning parameter, the analysis of predicted gene expression takes ∼33 s to 3.14 min (in a single core) to run when the memory ranges from 2.4 GB to 13.4 GB. For PCa, the disease aggressiveness, a more clinically relevant outcome, needs to be studied for evaluating the clinical utility of such efforts. Beyond PCa, PRSs developed using sophisticated methods like ours could potentially have clinical utility for other disease prediction, and highlights the importance of more work in this area with the aim of decreasing disease public health burden.

It is well known that the incorporation of family history could substantially improve model performance for risk prediction of PCa [30, 31, 32, 33, 34]. Interestingly, in our study, we observed that our newly developed PRS showed significantly higher risk assessment power than family history. The overall NRI was 69.0% when adding the developed PRS to the baseline model compared with 12.5% by adding family history. This encouraging result highlights the potential of incorporating the developed PRS for the risk assessment of PCa.

Our developed PRS could potentially bring opportunities for reducing the public health burden of PCa, though further studies are needed to independently assess the clinical utility of such PRS and evaluate more clinically relevant outcomes of aggressive PCa. The more accurate prediction of subjects at high risks before the development of PCa may better guide screening strategies than current practice for identifying aggressive PCa. Importantly, there exists a striking difference in the absolute risk of developing PCa between subjects with high PRSs and those with low PRSs. For males with a PRS within the bottom 50% range, their absolute risk was lower than 1.7%. In contrast, males with a PRS within the top 5% range had an absolute risk of 9.5%. These findings are very encouraging for developing screening strategies for targeting such high‐risk subjects. They could also facilitate the design of more efficient studies for identifying effective biomarkers for aggressive PCa early detection. Individuals who stay healthy despite a high PRS‐estimated risk or develop PCa despite a low PRS‐estimated risk may be of particular interest. Investigations on the discordance between PRS‐estimated risk and PCa status in these individuals may gain additional insights into the biological mechanisms of PCa, such as identifying rare variants with large effects missed by the PRS or gene‐environmental interactions in conventional study settings. Our innovative approach, including the revised P + T method for handling genetically imputed transcriptome and methylome information, and the novel integrative design to capture information from multiple methods can also be used to develop improved PRS for other complex diseases, beyond the scope of PCa. The calculation of risks of other diseases for each individual would have substantial utility in better guiding prevention or screening strategies, posing substantial public health implications to reduce disease burdens.

There are several limitations of the current study. First, the UK Biobank is not a representative sample of the whole UK population, and participants in the UK Biobank study are generally healthier than the general UK population. For example, compared with the general population, UK Biobank study participants were less likely to be obese and had fewer self‐reported health conditions [35, 36]. Therefore, our study may have underestimated the population‐level lifetime PCa risk. However, this may be alleviated by recalibration. Second, in this study, we focused on a European population. The developed PRS may not necessarily be generalizable to other ethnic groups. Future studies focusing on other non‐European ethnic groups would be needed to better serve underrepresented populations. Third, integrating genetic information related to other cancers [37] or other functional regulatory information [20] may further improve the prediction accuracy, which warrants further investigation. Fourth, in the current study, we investigated PRS that incorporate information of genetically predicted gene expression in prostate tissue and blood and DNA methylation levels in the blood. Future work incorporating information of genetically predicted DNA methylation levels in prostate tissue may further improve the model performance, particularly as it could potentially capture more information of prostate tissue‐specific signals.

## 5 CONCLUSIONS

In conclusion, we developed and validated a new PRS, which may have improved utility in predicting the risk of developing PCa in European males.

6

### DISCLOSURE OF POTENTIAL CONFLICTS OF INTEREST

No potential conflicts of interest were disclosed by the authors.

### AUTHOR CONTRIBUTIONS

Concept and design: LW, CW.

Acquisition, analysis, or interpretation of data: CW, JZ, AK, XT, QL, TRR, WP, LW.

Drafting of the manuscript: LW, CW, JZ.

Critical revision of the manuscript for important intellectual content: AK, XT, QL, JYP, LW, GG, HWD, YY, KEK, TRR, JL, WZ, WP, DVC, CAH.

Statistical analysis: CW, LW.

Obtained funding: CW, LW.

Administrative, technical, or material support: CW, LW.

Supervision: LW, CW.

## Supporting information

Figure S1‐oS3

Table S1–S3

## Data Availability

The summary statistics of genome‐wide association studies of prostate cancer in the PRACTICAL consortium are available at http://practical.icr.ac.uk/blog/?page_id=8164. The UK Biobank is an open‐access resource, available at https://www.ukbiobank.ac.uk/researchers/. This research was conducted with approved access to UK Biobank data under application numbers 48240 and 53866.
