# Supplementary material for: Novel strategy for disease risk prediction incorporating predicted gene expression and DNA methylation data: a multi‐phased study of prostate cancer
Source: Cancer Commun (Lond). 2021 Sep 14;41(12):1387–97. doi: 10.1002/cac2.12205 (PMC8696216; doi:10.1002/cac2.12205)
Supplement: Supplementary file 1 — Figure S1‐oS3 [file CAC2-41-1387-s002.docx]

**Supplementary Figures**

**Supplementary Figure S1. Correlation of PRSs constructed by different methods in the combining step using data of 1,467 prevalent PCa cases and 1,458 controls.** MethylationNG, Expression_Blood, and Expression_Prostate stand for the PRSs built by the revised pruning and thresholding strategy with predicted DNA methylation levels in blood, predicted gene expression levels in blood and in prostate tissues, respectively. PT and LDpredfunct stand for P+T and LDpred-funct. Abbreviations: PRSs, polygenic risk scores; PCa, prostate cancer.

**Supplementary Figure S2.** Calibration plots for polygenic risk scores (PRSs) for prostate cancer (PCa), using the testing data of 4,832 incident PCa cases and 142,869 controls. Recalibration was performed by fitting the predicted log hazard ratio as a covariate in a Cox model with a re-estimated baseline risk. PRS + Age and PRS represent our novel PRS plus baseline model with and without age, respectively. P_GND_ is the P value calculated by the Greenwood-Nam-D’Agostino test, a test that evaluates the null hypothesis that the observed and expected probabilities are identical in each group. $\beta$ is the calibration slope, and $\beta=1$ indicates a perfect calibration.

**Supplementary Figure S3.** Changes in predicted probabilities (in percentage) of the recalibrated model with baseline covariates after the addition of the polygenic risk score (PRS) for prostate cancer (PCa). To improve the visibility, a random draw of 1% of the participants is shown on the scatter plot.
